# 2-Carb­oxy-1-(3-nitro­phen­yl)ethanaminium perchlorate

**DOI:** 10.1107/S1600536809052167

**Published:** 2010-01-16

**Authors:** Wen-Xian Liang

**Affiliations:** a Ordered Matter Science Research Center, College of Chemistry and Chemical Engineering, Southeast University, Nanjing 210096, People’s Republic of China

## Abstract

In the title compound, C_9_H_11_N_2_O_4_^+^·ClO_4_^−^, the organic cations form centrosymmetric dimers *via* a pair of O—H⋯O hydrogen bonds between the carboxyl groups. In the crystal, N—H⋯O inter­actions between the protonated amine group and the perchlorate anions and the nitro group connect the components into a two-dimensional network parallel to (001).

## Related literature

For methods of preparation of β-amino acids, see: Cohen *et al.* (2002 ▶); Qu *et al.* (2004 ▶).
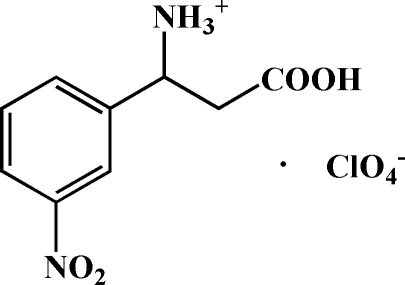


## Experimental

### 

#### Crystal data

C_9_H_11_N_2_O_4_^+^·ClO_4_^−^
*M* *_r_* = 310.65
Triclinic, 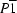

*a* = 7.4737 (10) Å
*b* = 7.7676 (8) Å
*c* = 11.8234 (11) Å
α = 94.973 (5)°
β = 99.093 (4)°
γ = 115.574 (9)°
*V* = 602.04 (12) Å^3^
*Z* = 2
Mo *K*α radiation
μ = 0.36 mm^−1^
*T* = 93 K
0.45 × 0.30 × 0.15 mm

#### Data collection

Rigaku SCXmini diffractometer
Absorption correction: multi-scan (*CrystalClear*; Rigaku, 2005 ▶) *T*_min_ = 0.882, *T*_max_ = 0.950
6395 measured reflections
2737 independent reflections
1732 reflections with *I* > 2σ(*I*)
*R*_int_ = 0.049

#### Refinement

*R*[*F* ^2^ > 2σ(*F* ^2^)] = 0.030
*wR*(*F* ^2^) = 0.083
*S* = 1.08
2737 reflections
183 parameters
H-atom parameters constrained
Δρ_max_ = 0.41 e Å^−3^
Δρ_min_ = −0.38 e Å^−3^

### 

Data collection: *CrystalClear* (Rigaku 2005 ▶); cell refinement: *CrystalClear*; data reduction: *CrystalClear*; program(s) used to solve structure: *SHELXS97* (Sheldrick, 2008 ▶); program(s) used to refine structure: *SHELXL97* (Sheldrick, 2008 ▶); molecular graphics: *SHELXTL* (Sheldrick, 2008 ▶); software used to prepare material for publication: *PRPKAPPA* (Ferguson, 1999 ▶).

## Supplementary Material

Crystal structure: contains datablocks I, New_Global_Publ_Block. DOI: 10.1107/S1600536809052167/gk2243sup1.cif

Structure factors: contains datablocks I. DOI: 10.1107/S1600536809052167/gk2243Isup2.hkl

Additional supplementary materials:  crystallographic information; 3D view; checkCIF report

## Figures and Tables

**Table 1:** Hydrogen-bond geometry (Å, °)

*D*—H⋯*A*	*D*—H	H⋯*A*	*D*⋯*A*	*D*—H⋯*A*
O3—H3⋯O4^i^	0.82	1.82	2.6381 (14)	172
N2—H2*D*⋯O5^ii^	0.89	2.30	3.0542 (17)	142
N2—H2*D*⋯O1^iii^	0.89	2.26	2.9200 (17)	130
N2—H2*C*⋯O8^iv^	0.89	2.06	2.9159 (17)	162
N2—H2*C*⋯O4	0.89	2.45	2.9317 (16)	114
N2—H2*B*⋯O7	0.89	2.57	3.1795 (18)	126
N2—H2*B*⋯O6	0.89	2.09	2.9720 (17)	172

Symmetry codes: (i) 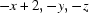; (ii) 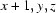; (iii) 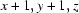; (iv) 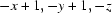.

**Fig. 1.**
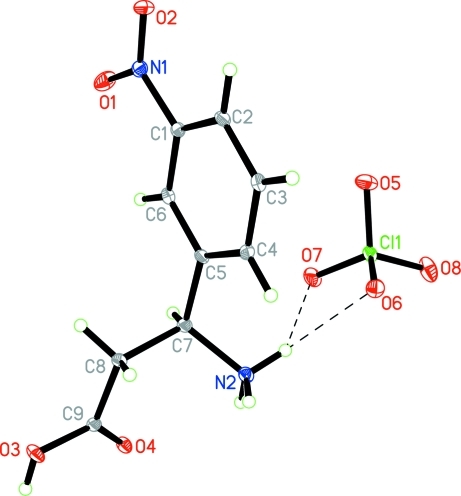
Cation and anion in the title compound with the displacement ellipsoids drawn at the 30% probability level. Intramolecular hydrogen bonds are shown as dashed lines.

**Fig. 2.**
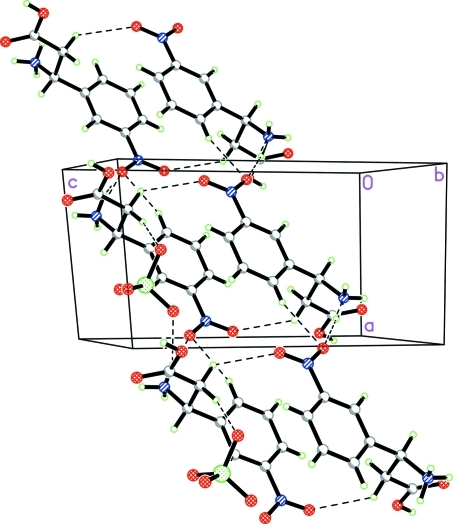
Packing diagram of the title compound showing hydrogen bonding interactions. Hydrogen bonds are shown as dashed lines.

## supplementary crystallographic information

### Comment

β-Amino acids are important molecules due to their pharmacological properties. Recently, there have been an increased interest in the enantiomeric preparation of β-amino acids as precursors for the synthesis of novel biologically active compounds (Cohen *et al.*, 2002). In addition, β-amino acids are attractive ligands for use in the generation of polar coordination polymers, especially when one considers the ferroelectric compounds (Qu *et al.*, 2004).

The molecular structure of the title compound C_9_H_11_N_2_O_4_^+^.ClO_4_^-^ is shown in Fig. 1. The crystal is stabilized by intermolecucar hydrogen bonds of N—H···O, C—H···O, O—H···O type (Table 1) that connect neighbouring cations and anions, resulting in a two-dimensional network shown in Fig. 2).

### Experimental

Under nitrogen protection, 3-nitrobenzaldehyde (4.53 g, 30 mmol), malonic acid (5.0 g, 48 mmol) and ammonium acetate (6.0 g, 78 mmol) were added to a flask and refluxed for 12 h yielding a white precipitate. After being cooled to room temperature, the solution was filtered and the 3-amino-3-(3-nitrophenyl)propanoic acid was obtained, it was dissolved in ethanol and perchloric acid. After slowly evaporating over a period of 3 d, colorless prism crystals of the title compound suitable for diffraction studies were isolated.

### Refinement

Positional parameters of all H atoms were calculated geometrically and H atoms were allowed to ride on their parent atoms, with C—H = 0.93 to 0.97 Å, N—H = 0.89 Å, O—H = 0.82 Å, and with *U*_iso_(H) = 1.2 *U*_eq_o(C) or 1.5 *U*_eq_(N,*O*).

### Crystal data

**Table:**

C_9_H_11_N_2_O_4_^+^·ClO_4_^−^	*Z* = 2
*M**_r_* = 310.65	*F*(000) = 320
Triclinic, *P*1	*D*_x_ = 1.713 Mg m^−^^3^
Hall symbol: -P 1	Mo *K*α radiation, λ = 0.71073 Å
*a* = 7.4737 (10) Å	Cell parameters from 1721 reflections
*b* = 7.7676 (8) Å	θ = 3.1–27.5°
*c* = 11.8234 (11) Å	µ = 0.36 mm^−^^1^
α = 94.973 (5)°	*T* = 93 K
β = 99.093 (4)°	Prism, colorless
γ = 115.574 (9)°	0.45 × 0.30 × 0.15 mm
*V* = 602.04 (12) Å^3^	

### Data collection

**Table:**

Rigaku SCXmini diffractometer	2737 independent reflections
Radiation source: fine-focus sealed tube	1732 reflections with *I* > 2σ(*I*)
graphite	*R*_int_ = 0.049
Detector resolution: 13.6612 pixels mm^-1^	θ_max_ = 27.5°, θ_min_ = 2.9°
ω and φ scan	*h* = −9→9
Absorption correction: multi-scan (*CrystalClear*; Rigaku, 2005)	*k* = −10→10
*T*_min_ = 0.882, *T*_max_ = 0.950	*l* = −15→15
6395 measured reflections	

### Refinement

**Table:**

Refinement on *F*^2^	Secondary atom site location: difference Fourier map
Least-squares matrix: full	Hydrogen site location: inferred from neighbouring sites
*R*[*F*^2^ > 2σ(*F*^2^)] = 0.030	H-atom parameters constrained
*wR*(*F*^2^) = 0.083	*w* = 1/[σ^2^(*F*_o_^2^) + (0.0372*P*)^2^ + 0.395*P*] where *P* = (*F*_o_^2^ + 2*F*_c_^2^)/3
*S* = 1.08	(Δ/σ)_max_ = 0.001
2737 reflections	Δρ_max_ = 0.41 e Å^−^^3^
183 parameters	Δρ_min_ = −0.38 e Å^−^^3^
0 restraints	Extinction correction: *SHELXL*
Primary atom site location: structure-invariant direct methods	Extinction coefficient: 0.0014 (2)

### Special details

**Table:**

Geometry. All e.s.d.'s (except the e.s.d. in the dihedral angle between two l.s. planes) are estimated using the full covariance matrix. The cell e.s.d.'s are taken into account individually in the estimation of e.s.d.'s in distances, angles and torsion angles; correlations between e.s.d.'s in cell parameters are only used when they are defined by crystal symmetry. An approximate (isotropic) treatment of cell e.s.d.'s is used for estimating e.s.d.'s involving l.s. planes.
Refinement. Refinement of *F*^2^ against ALL reflections. The weighted *R*-factor *wR* and goodness of fit *S* are based on *F*^2^, conventional *R*-factors *R* are based on *F*, with *F* set to zero for negative *F*^2^. The threshold expression of *F*^2^ > σ(*F*^2^) is used only for calculating *R*-factors(gt) *etc*. and is not relevant to the choice of reflections for refinement. *R*-factors based on *F*^2^ are statistically about twice as large as those based on *F*, and *R*- factors based on ALL data will be even larger.

### Fractional atomic coordinates and isotropic or equivalent isotropic displacement parameters (Å^2^)

**Table:**

	*x*	*y*	*z*	*U*_iso_*/*U*_eq_	
O1	0.03107 (18)	−0.28766 (17)	0.32513 (10)	0.0261 (3)	
O2	0.04653 (17)	−0.16514 (17)	0.49951 (10)	0.0222 (2)	
O3	0.98215 (17)	−0.04224 (17)	0.14192 (9)	0.0188 (2)	
H3	1.0329	−0.0530	0.0868	0.028*	
O4	0.82886 (16)	0.08503 (15)	0.02079 (9)	0.0166 (2)	
N1	0.11075 (18)	−0.15634 (18)	0.40993 (11)	0.0163 (3)	
N2	0.74545 (19)	0.36296 (18)	0.15908 (10)	0.0155 (2)	
H2D	0.8712	0.4242	0.2025	0.023*	
H2B	0.6791	0.4320	0.1712	0.023*	
H2C	0.7504	0.3487	0.0843	0.023*	
C1	0.2944 (2)	0.0167 (2)	0.40370 (12)	0.0136 (3)	
C2	0.3868 (2)	0.1673 (2)	0.49778 (12)	0.0142 (3)	
H2A	0.3309	0.1630	0.5630	0.017*	
C3	0.5666 (2)	0.3255 (2)	0.49154 (12)	0.0150 (3)	
H3A	0.6328	0.4284	0.5536	0.018*	
C4	0.6474 (2)	0.3303 (2)	0.39298 (12)	0.0146 (3)	
H4	0.7681	0.4360	0.3899	0.018*	
C5	0.5485 (2)	0.1772 (2)	0.29839 (12)	0.0137 (3)	
C6	0.3711 (2)	0.0183 (2)	0.30398 (12)	0.0139 (3)	
H6	0.3046	−0.0853	0.2423	0.017*	
C7	0.6363 (2)	0.1664 (2)	0.19179 (12)	0.0142 (3)	
H7	0.5242	0.0828	0.1264	0.017*	
C8	0.7776 (2)	0.0740 (2)	0.21606 (12)	0.0146 (3)	
H8A	0.8887	0.1565	0.2808	0.018*	
H8B	0.7035	−0.0492	0.2399	0.018*	
C9	0.8654 (2)	0.0398 (2)	0.11572 (12)	0.0136 (3)	
Cl1	0.33694 (5)	0.50865 (5)	0.14980 (3)	0.01593 (11)	
O5	0.18796 (17)	0.43639 (18)	0.21983 (10)	0.0261 (3)	
O7	0.31799 (18)	0.35386 (17)	0.06549 (11)	0.0270 (3)	
O8	0.3095 (2)	0.6550 (2)	0.09212 (10)	0.0302 (3)	
O6	0.53816 (16)	0.59598 (16)	0.22457 (10)	0.0209 (2)	

### Atomic displacement parameters (Å^2^)

**Table:**

	*U*^11^	*U*^22^	*U*^33^	*U*^12^	*U*^13^	*U*^23^
O1	0.0238 (6)	0.0184 (6)	0.0237 (6)	−0.0008 (5)	0.0036 (5)	0.0009 (5)
O2	0.0208 (5)	0.0255 (6)	0.0254 (6)	0.0112 (5)	0.0135 (5)	0.0098 (5)
O3	0.0227 (5)	0.0280 (6)	0.0151 (5)	0.0186 (5)	0.0074 (4)	0.0048 (4)
O4	0.0196 (5)	0.0206 (5)	0.0144 (5)	0.0130 (4)	0.0051 (4)	0.0031 (4)
N1	0.0138 (6)	0.0171 (6)	0.0197 (6)	0.0082 (5)	0.0035 (5)	0.0062 (5)
N2	0.0186 (6)	0.0164 (6)	0.0148 (5)	0.0099 (5)	0.0060 (5)	0.0034 (5)
C1	0.0119 (6)	0.0146 (7)	0.0169 (6)	0.0079 (5)	0.0031 (5)	0.0049 (5)
C2	0.0155 (6)	0.0176 (7)	0.0140 (6)	0.0105 (6)	0.0056 (5)	0.0047 (5)
C3	0.0162 (6)	0.0136 (7)	0.0156 (6)	0.0083 (5)	0.0016 (5)	−0.0001 (5)
C4	0.0121 (6)	0.0124 (6)	0.0193 (7)	0.0050 (5)	0.0045 (5)	0.0032 (5)
C5	0.0153 (6)	0.0153 (7)	0.0145 (6)	0.0098 (5)	0.0054 (5)	0.0032 (5)
C6	0.0148 (6)	0.0140 (6)	0.0145 (6)	0.0086 (5)	0.0017 (5)	0.0012 (5)
C7	0.0144 (6)	0.0135 (6)	0.0146 (6)	0.0061 (5)	0.0047 (5)	0.0007 (5)
C8	0.0167 (6)	0.0153 (6)	0.0148 (6)	0.0084 (5)	0.0073 (5)	0.0039 (5)
C9	0.0122 (6)	0.0119 (6)	0.0157 (6)	0.0045 (5)	0.0041 (5)	0.0010 (5)
Cl1	0.01637 (17)	0.01607 (18)	0.01561 (17)	0.00828 (13)	0.00303 (13)	−0.00028 (13)
O5	0.0183 (5)	0.0306 (6)	0.0253 (6)	0.0070 (5)	0.0087 (5)	−0.0011 (5)
O7	0.0272 (6)	0.0203 (6)	0.0294 (6)	0.0086 (5)	0.0082 (5)	−0.0080 (5)
O8	0.0498 (8)	0.0372 (7)	0.0175 (5)	0.0332 (6)	0.0043 (5)	0.0059 (5)
O6	0.0167 (5)	0.0202 (5)	0.0231 (5)	0.0072 (4)	0.0013 (4)	0.0023 (4)

### Geometric parameters (Å, °)

**Table:**

O1—N1	1.2288 (17)	C3—H3A	0.9300
O2—N1	1.2255 (17)	C4—C5	1.400 (2)
O3—C9	1.3011 (18)	C4—H4	0.9300
O3—H3	0.8200	C5—C6	1.384 (2)
O4—C9	1.2296 (18)	C5—C7	1.5217 (18)
N1—C1	1.4670 (18)	C6—H6	0.9300
N2—C7	1.5075 (18)	C7—C8	1.519 (2)
N2—H2D	0.8900	C7—H7	0.9800
N2—H2B	0.8900	C8—C9	1.5000 (18)
N2—H2C	0.8900	C8—H8A	0.9700
C1—C2	1.384 (2)	C8—H8B	0.9700
C1—C6	1.3889 (19)	Cl1—O7	1.4354 (11)
C2—C3	1.394 (2)	Cl1—O5	1.4396 (12)
C2—H2A	0.9300	Cl1—O8	1.4455 (12)
C3—C4	1.391 (2)	Cl1—O6	1.4486 (11)
			
C9—O3—H3	109.5	C5—C6—C1	118.60 (13)
O2—N1—O1	123.17 (13)	C5—C6—H6	120.7
O2—N1—C1	118.90 (12)	C1—C6—H6	120.7
O1—N1—C1	117.91 (12)	N2—C7—C8	111.06 (11)
C7—N2—H2D	109.5	N2—C7—C5	111.99 (11)
C7—N2—H2B	109.5	C8—C7—C5	108.58 (11)
H2D—N2—H2B	109.5	N2—C7—H7	108.4
C7—N2—H2C	109.5	C8—C7—H7	108.4
H2D—N2—H2C	109.5	C5—C7—H7	108.4
H2B—N2—H2C	109.5	C9—C8—C7	115.16 (12)
C2—C1—C6	123.03 (13)	C9—C8—H8A	108.5
C2—C1—N1	119.16 (12)	C7—C8—H8A	108.5
C6—C1—N1	117.78 (13)	C9—C8—H8B	108.5
C1—C2—C3	117.80 (13)	C7—C8—H8B	108.5
C1—C2—H2A	121.1	H8A—C8—H8B	107.5
C3—C2—H2A	121.1	O4—C9—O3	125.13 (13)
C4—C3—C2	120.38 (13)	O4—C9—C8	122.98 (13)
C4—C3—H3A	119.8	O3—C9—C8	111.90 (12)
C2—C3—H3A	119.8	O7—Cl1—O5	110.51 (7)
C3—C4—C5	120.49 (13)	O7—Cl1—O8	110.15 (7)
C3—C4—H4	119.8	O5—Cl1—O8	109.25 (8)
C5—C4—H4	119.8	O7—Cl1—O6	109.10 (7)
C6—C5—C4	119.70 (13)	O5—Cl1—O6	109.02 (7)
C6—C5—C7	116.93 (12)	O8—Cl1—O6	108.78 (8)
C4—C5—C7	123.14 (12)		
			
O2—N1—C1—C2	−1.71 (19)	C7—C5—C6—C1	175.40 (12)
O1—N1—C1—C2	179.73 (13)	C2—C1—C6—C5	0.3 (2)
O2—N1—C1—C6	176.20 (12)	N1—C1—C6—C5	−177.56 (12)
O1—N1—C1—C6	−2.37 (19)	C6—C5—C7—N2	148.40 (12)
C6—C1—C2—C3	−0.9 (2)	C4—C5—C7—N2	−37.23 (18)
N1—C1—C2—C3	176.88 (12)	C6—C5—C7—C8	−88.61 (15)
C1—C2—C3—C4	0.5 (2)	C4—C5—C7—C8	85.76 (16)
C2—C3—C4—C5	0.6 (2)	N2—C7—C8—C9	−60.66 (16)
C3—C4—C5—C6	−1.2 (2)	C5—C7—C8—C9	175.79 (12)
C3—C4—C5—C7	−175.45 (13)	C7—C8—C9—O4	0.9 (2)
C4—C5—C6—C1	0.8 (2)	C7—C8—C9—O3	−179.00 (12)

### Hydrogen-bond geometry (Å, °)

**Table:**

*D*—H···*A*	*D*—H	H···*A*	*D*···*A*	*D*—H···*A*
C8—H8B···O6^i^	0.97	2.46	3.3742 (19)	157
C8—H8A···O2^ii^	0.97	2.56	3.3053 (19)	134
C6—H6···O8^i^	0.93	2.59	3.4263 (19)	151
C4—H4···O1^iii^	0.93	2.48	3.3858 (19)	163
O3—H3···O4^iv^	0.82	1.82	2.6381 (14)	172
N2—H2D···O5^v^	0.89	2.30	3.0542 (17)	142
N2—H2D···O1^iii^	0.89	2.26	2.9200 (17)	130
N2—H2C···O8^vi^	0.89	2.06	2.9159 (17)	162
N2—H2C···O4	0.89	2.45	2.9317 (16)	114
N2—H2B···O7	0.89	2.57	3.1795 (18)	126
N2—H2B···O6	0.89	2.09	2.9720 (17)	172

Symmetry codes: (i) *x*, *y*−1, *z*; (ii) −*x*+1, −*y*, −*z*+1; (iii) *x*+1, *y*+1, *z*; (iv) −*x*+2, −*y*, −*z*; (v) *x*+1, *y*, *z*; (vi) −*x*+1, −*y*+1, −*z*.
